# DNA Damage Stress Response and Follicle Activation: Signaling Routes of Mammalian Ovarian Reserve

**DOI:** 10.3390/ijms232214379

**Published:** 2022-11-19

**Authors:** Stefania Gonfloni, Carla Jodice, Bianca Gustavino, Elvia Valentini

**Affiliations:** 1 Department of Biology, University of Rome Tor Vergata, Via Della Ricerca Scientifica, 00133 Rome, Italy; 2 PhD Program in Cellular and Molecular Biology, 00133 Rome, Italy

**Keywords:** DNA damage response, chemotherapy, ovarian reserve, follicle activation, p53

## Abstract

Chemotherapy regimens and radiotherapy are common strategies to fight cancer. In women, these therapies may cause side effects such as premature ovarian insufficiency (POI) and infertility. Clinical strategies to protect the ovarian reserve from the lethal effect of cancer therapies needs better understanding of the mechanisms underlying iatrogenic loss of follicle reserve. Recent reports demonstrate a critical role for p53 and CHK2 in the oocyte response to different DNA stressors, which are commonly used to treat cancer. Here we review the molecular mechanisms underlying the DNA damage stress response (DDR) and discuss crosstalk between DDR and signaling pathways implicated in primordial follicle activation.

## 1. Introduction

The mammalian ovary is the primary female reproductive organ involved in oocyte maturation and in the synthesis and secretion of sex steroid hormones, estrogens and progesterone, which are crucial for female fertility [1]. The cortex region of the human ovary possesses a finite pool of primordial follicles, the ovarian reserve [2], whose number is set before birth. Each primordial follicle contains only one immature and quiescent oocyte, surrounded by a layer of flattened pre-granulosa cells. Quiescence is a state of reversible growth arrest at diplotene of the meiotic prophase I. Primordial oocytes produced at birth can remain quiescent for the entire female reproductive life. The maintenance of the quiescent state is essential for chromosomal stability of oocytes and for preservation of the primordial follicles during reproductive life.

The activation of mammalian primordial follicles consists in an irreversible mechanism where a primordial follicle leaves the quiescence status and starts oocyte growth [3]. Follicle activation implies several changes of granulosa cells (from flat to cuboidal shape, marking the beginning of the primary follicle), an increase in cell number in tandem with a strict reorganization within the follicle. All these modifications are crucial for the development and release of a mature egg competent for fertilization [4]. Strategically positioned around the oocyte, granulosa cells are able to communicate through a bidirectional somatic cell–oocyte signaling pathway [5]. Such a bidirectional exchange guides the primordial follicle into a cohort of growing follicles from which only one antral follicle, called the “dominant follicle”, is chosen to ovulate in a competent cumulus-oocyte complex (COC) into the Fallopian tube. Of note, the competence of the oocyte is defined as the capability of the oocyte to conclude meiosis and go through fertilization, embryogenesis, and final development [6]. Yet, the rest of the growing follicle pool degenerates and dies in a process called “atresia”, which means that a woman, every month, loses many immature eggs (6–8) per menstrual cycle.

Recent studies have demonstrated the adverse effects of cancer therapies on the reproductive potential of young women [7]. Exposure to chemotherapeutic agents is responsible for the degeneration of the ovarian reserve, resulting in premature ovarian insufficiency (POI) [8] and increased risk of infertility. In recent years, the increase in the survival rate of female patients undergoing chemotherapy treatments boosts the need to clarify the molecular mechanisms underlying POI. In this regard, many studies concerning the complex signaling pathways involved in follicular quiescence and in survival are emerging. These involve a multidisciplinary area of investigations, also including the implementation of biomaterials for tissue engineering [9].

Chemotherapy drugs can damage the ovary through different mechanisms [10]. In mice, compelling evidence indicates that genotoxic drugs induce ovarian reserve depletion. However, it is still debated whether this loss is due to an accelerated follicle activation, to a direct execution of DDR in reserve oocytes, or, most likely, to a crosstalk between the two signaling cascades. In this article, we focus on these two key pathways in the maintenance of the primordial follicle pool.

## 2. Primordial Follicle Activation

**Figure 1:**
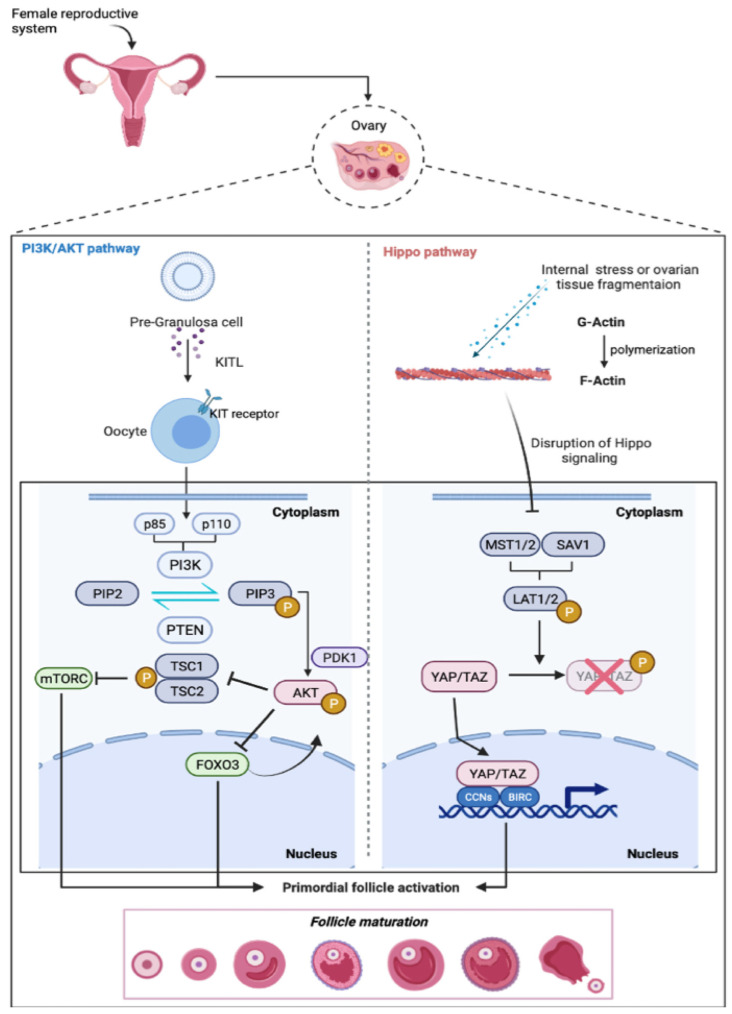
**PI3K-PTEN-AKT-FOXO3-mTOR and the Hippo signaling pathways as primary regulators of primordial follicle activation**. At the top of the figure, the female reproductive system (ovary and follicle enlarged) is shown, as well as a schematic representation of follicle activation steps. In the PI3K/AKT pathway, PI3K is activated by the binding of the KIT ligand (KITL), released by pre-granulosa cells (GCs) to the KIT/tyrosine kinase receptor on the oocyte. PI3K phosphorylates PIP2 into PIP3, promoting PDK1 recruitment to the membrane. In turn, PDK1 activates Akt, which moves to the nucleus and phosphorylates the FOXO3 transcription factor. On the contrary, PTEN dephosphorylates PIP3 to PIP2 while preventing the activation of the follicle. (B) Hippo pathway: Internal stresses or ovarian tissue fragmentations lead to the polymerization of globular actin (G-actin) to filamentous actin (F-Actin), causing disruption to the Hippo signaling pathway, which results in the expression of MTS1/2 and SAV1 complex. The latter is able to phosphorylate LATS1/2 (large tumor suppressor 1 and 2). LATS1/2 prevent the phosphorylation of YAP and TAZ, promoting their translocation into the nucleus. YAP/TAZ stimulate the transcription of downstream growth factors stimulators such as 1 CYR61/CCN1, CTGF/CCN2, and BIRC genes, resulting in primordial follicle activation and development.

PI3K-PTEN-AKT-FOXO3-mTOR and the Hippo signaling pathways are considered two primary regulators of primordial follicle activation at the time of the recruitment into the growing follicle pool (both pathways are represented in Figure 1). Specifically, the protein-kinase B (Akt) pathway intercedes between the primordial follicle activation through the phosphoinositide 3-kinase-phosphatase and the tensin homolog (PI3K-PTEN) signaling cascade. In particular, the PI3K pathway controls cellular proliferation, cell cycle entrance, and cell survival in the human ovary, while the PTEN protein negatively regulates PI3K [11,12]; the Hippo pathway is mainly involved in the conservation of the optimal size of organs through growth inhibition [13].

### 2.1. The PI3K-PTEN-Akt Signaling Axis

The activation of the PI3K complex is triggered by the binding of receptor tyrosine kinase (KITL) ligand, released by pre-granulosa cells (GCs), to its receptor (KIT) on the oocyte. Once activated, PI3K modifies phosphatidylinositol-4,5-bisphosphate (PIP2) into phosphatidylinositol-3,4,5-triphosphate (PIP3). The latter drives the recruitment of both pyruvate dehydrogenase kinase 1 PDK1 and Akt at the membrane, where PDK1 phosphorylation activates Akt. In turn, Akt moves into the nucleus where it phosphorylates the forkhead box O3 (FOXO3) transcription factor [14]. In primordial murine follicles, nuclear FOXO3 preserves follicular dormancy [15], while Akt-mediated phosphorylation promotes FOXO3 export into the cytoplasm. Indeed, in mice, p-FOXO3 (Akt-phosphorylated on residual Thr32) was detected in the cytoplasm of the growing oocyte [16]. Moreover, a mouse model lacking FOXO3 (Foxo3-/-) presents an overall follicular activation, resulting in a premature infertility [17]. The PTEN signaling cascade has a key role in the maintenance of follicular dormancy [18]. PTEN, acting on PIP2, prevents follicular activation through inhibition of the Akt–FOXO3 signaling axis. Compelling evidence indicates that lack of PTEN or of 3-phosphoinositide-dependent protein kinase-1 (Pdk1) gene results in activation of the whole pool of primordial follicles [19,20], suggesting that the PI3K–PTEN signaling cascade deeply regulates the initial recruitment of primordial follicles [21]. In adult mice, lack of PTEN has no effect on the primordial follicle pool, follicular development, ovulation, or long-term fertility [22]. In addition, Liu and colleagues reported that PTEN inhibitor could activate primordial follicles derived from human ovarian cortex strips. Such activated follicles reached maturation following xenotransplantation into immunodeficient mice [23]. Again, McLaughlin and colleagues reported an increase in primordial follicle activation after treatment with PTEN inhibitor, together with a compromised development of growing follicles in the ovary [24].

Akt is also able to phosphorylate the tuberous sclerosis 2 protein (TSC2), which forms a heterodimer complex with tuberous sclerosis 1 protein (TSC1). In cells, the TSC1/TSC2 complex negatively regulates a target of rapamycin complex 1 (mTORC1) [25], the latter is a serine/threonine kinase implicated in the regulation of cell growth and metabolism. mTORC1 can activate p70 S6 kinase 1 (S6K1) and the ribosomal protein S6 (rpS6), while inactivating the eukaryotic translation initiation factor 4E (4E-BPs). Adhikari and colleagues in their studies reported that in a conditional knockout mouse model for Tsc1 and Tsc2, all primordial follicles are overall activated at puberty, resulting in premature ovarian insufficiency [26]. Furthermore, Tsc1 deletion in pre-granulosa cells can end in a global primordial follicle activation [27]. This observation suggests that the TSC1/TSC2 complex is extremely important in the regulation of follicular activation for the ovarian reserve. An increase in the number of antral/pre-ovulatory follicles was also found after treatment of both mTOR and Akt signaling activators compared to the treatment with only Akt activators [28]. Interestingly, Sun and colleagues proposed an approach using both stimulators of mammalian target of rapamycin (mTOR) and PI3K. Through histological analysis, they showed that both compounds are able to increase the growth of human follicles in ovarian cortex [29].

### 2.2. Hippo Signaling

As mentioned before, the Hippo signaling pathway (also known as the Salvador (Sav)-Warts (Wts)-Hippo (Hpo) (SWH) pathway) has a crucial role in controlling cell growth and organ size [30]. The Hippo pathway consists of a serine/threonine protein kinase signaling cascade, resulting in phosphorylation and inactivation of two major transcriptional coactivators, Yes-associated protein (YAP) and transcriptional coactivators PDZ-binding motif (TAZ) [31]. The Hippo signaling pathway can also suppresses follicular reserve activation. Recent studies reported that an increased internal stress or an ovarian tissue fragmentation can promote the polymerization of globular actin (G-Actin) into filamentous actin (F-actin). This caused a perturbation of the Hippo signaling pathway and expression of mammalian sterile 20-like (MTS1/2) and Salvador family WW domain containing protein 1 (SAV1) complex [32,33], which in turn phosphorylates large tumor-suppressor 1 and 2 (LATS1/2). LATS1/2 inhibited the phosphorylation of YAP and TAZ. When not phosphorylated, YAP/TAZ moves into the nucleus and stimulates the transcription of cysteine-rich protein 61/cellular communicator network factor 1 (CYR61/CCN1), connective tissue growth factors/cellular communicator network factor 2 (CTGF/CCN2), and baculoviral inhibitors of apoptosis repeat containing (BIRC) genes. Such events trigger primordial follicle activation and development [34]. Jaspakinolide (JASP) and sphingosine-1-phosphate (S1P), two actin polymerization-enhancing drugs, cause the Hippo signaling disorder, as indicated by a rise in both ovarian graft weights and the number of secondary follicles [35]. Furthermore, during follicular development, an increased expression of YAP1 is concomitant with reduced MTS1 expression [36].

As mentioned above, the ovarian reserve is composed of a finite pool of primordial follicles whose number is established before birth. This ovarian reserve represents the capability to generate offspring [37]. In women, with increasing age, the quality and the quantity of eggs begin to decline, accompanied by a gradual reduction of ovarian function. Age-related decline of the ovarian reserve is dependent on the microenvironment and the quality of stored oocytes [38]. In addition, the advanced age of women influences oocyte maturation, meiotic division, and embryo development [39]. Data from the literature show that metabolic disorders such as diabetes and acute inflammation, which include an increase in reactive oxygen species (ROS) levels, may have repercussion on female fertility [40]. Concerning glucose metabolism, it seems that the right intake of glucose by granulosa cells is decisive for primordial follicle activation. As shown by Xu and colleagues [41], in the mouse, glucose level influences the trigger of primordial follicle activation in vitro and in vivo through the AMPK/mTOR pathway. In fact, when the glucose concentration in culture growth medium or blood is lower than threshold levels, AMP-activated protein kinase (AMPK) level increases while the mTOR pathway is inactivated. This in turn preserves ovarian follicle quiescence. Likewise, in women, high levels of ROS in the follicular microenvironment have a severe impact on primordial follicles and ovarian function [42]. Besides, compelling evidence indicates that high ROS level may disrupt the Hippo signaling pathway and thereby the maintenance of follicle dormancy [43].

## 3. DNA Damage Stress Response and Primordial Follicle Growth

**Figure 2:**
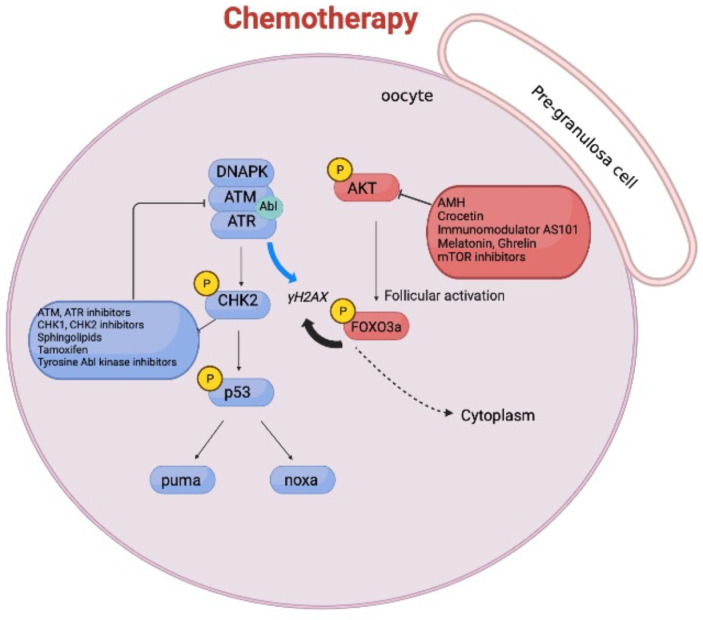
**Concomitant firing of both the follicular activation pathway and DDR.** Chemotherapy triggers both the follicular activation pathway and DDR. A hallmark of the follicular activation cascade is FOXO3 phosphorylation. Akt-mediated phosphorylation promotes the export of FOXO3 to the cytoplasm. DDR is induced by DNAPK/ATR/ATM (c-Abl tyrosine kinase is also involved in their activation), which in turn can activate the CHK2-p53 signaling cascade, promoting the transcription of PUMA and NOXA genes.

Cancer treatments with higher genotoxic potential, such as alkylating drugs, cyclophosphamide (Cy), and platinum-based complexes create DNA adducts that interfere with cell cycle progression and replication [44,45]. Chemotherapy drugs damage both healthy cells and the quiescent reserve oocytes. Recent findings suggest that chemotherapeutic treatments result in the activation of the PTEN-PI3K-Akt-mTOR signaling pathway, which ends in an accelerated follicular growth [46] called “burn out” [46,47]. Thus, primordial follicle depletion and the consequent loss of reproductive potential in the patients [48] can be due either to an accelerated follicle activation or to a direct activation of DDR in the nucleus of the reserve oocyte. In mice, we and other groups show that Cy administration induces cell death in the granulosa cells surrounding the growing follicles, as measured by TdT-mediated dUTP nick-end labeling (TUNEL) assay [49,50]. Follicular apoptosis, which reduces anti-Mullerian hormone (AMH) secretion, may trigger an upregulation of the PI3K-Akt-mTOR pathway in the pre-granulosa cells of primordial follicles. In line with this, in mice, compelling evidence demonstrates that AMH administration mitigates ovarian reserve loss induced by Cy [51,52]. The biochemical pathway that regulates primordial follicle activation in rodents is centered on translocation of FOXO3 from the nucleus to the cytoplasm [53]. Targeting the PI3K-PTEN-Akt-mTOR pathway to prevent FOXO3 nuclear shuttling has been considered as a means to protect ovaries following exposure to chemotherapy, and promising results using pharmacological inhibitors of mTOR are reported by several groups [27,54,55]. Chemotherapy may directly target resting primordial follicles embedded in avascular regions of the ovarian cortex. Immunofluorescence (IF) assays for cleaved poly ADP ribose polymerase (PARP) and for the histone variant H2AX, phosphorylated at Ser139 (γH2AX), indicate that reserve oocytes are damaged by chemotherapy. Within the reserve follicle, DDR is mediated by the activation of apical DDR kinases (DNA-PK, ATM), Check-point kinase 2 (CHK2), and p53, as revealed by IF assays using phosphor-specific antibodies. In addition, Cromatin Immunoprecipitation assay (ChIp) performed on ovaries collected from cyclophosphamide-injected pups shows that p53 drives the transcription of the pro-apoptotic gene PUMA ([50] see supplementary information). In mice, following cyclophosphamide treatments in vivo, we also find that reserve oocytes are positive for both p-Akt/p-FOXO3 and p-ATM/γH2AX expression ([50] see supplementary information). This indicates that DDR markers’ expression is concomitant with the activation of the PTEN-PI3K-Akt-mTOR-FOXO3 pathway. These findings suggest that follicle growth signals act in parallel with the activation of DDR (as it is proposed in Figure 2). Thus, combinations of inhibitors (recently reviewed [10]) for both pathways may protect more efficiently the ovarian reserve from genotoxic stressors.

## 4. Molecular Pathways Involved in Chemotherapy-Induced Ovarian Reserve Loss

Studies in mouse models indicate that TAp63alfa is expressed in primordial oocytes [56]. Excess reserve oocytes are detected in TAp63-null mice (within the first week after birth), suggesting a role of TAp63alfa in controlling the natural removal of damaged primordial follicles [57]. Following ionizing radiation (IR), ovaries from the same TAp63-null mice (P5) still have some reserve oocytes compared to control mice, 48 h after IR [58]. However, further studies in mice indicate that CHK2 and p53 both have crucial roles in the efficient removal of oocytes with unrepaired meiotic DNA double-strand breaks (DBSs) [59]. In addition, CHK2-mediated phosphorylation of p53 and of TAp63alfa (at S621 residue, S582 in human) are both induced in response to IR [59,60,61]. Recent reports suggest that following CHK2-mediated phosphorylation, the TAp63-tetramer may become a substrate of casein kinase (CK1), which in turn continues the TAp63 activation cascade [62]. This model for TAp63alfa activation is also proposed following exposure to diverse chemotherapeutic drugs [63] by using in vitro experiments. However, in these experiments, the authors did not report the status of activation of either p53 or CHK2. In addition, studies ex vivo of murine ovarian fragments and biochemical investigations performed in cell lines lacking p53 can only partially recapitulate the signaling pathways occurring in vivo in the ovary following chemotherapy. The use of gene deletion mouse models may be informative to define the mechanisms underlying the chemotherapy-induced ovarian reserve loss. However, such experimental evidence must be further confirmed either by using specific pharmacological inhibitors or through a transgenic mouse model carrying a single-point mutation with loss of function. In mice, loss of PUMA (in all cell types forming the ovary) protects the ovarian reserve during chemotherapy and preserves fertility [64]. In the same manuscript, the authors report that primordial follicles from TAp63-null mice are protected following cisplatin but not cyclophosphamide, suggesting mechanistic differences in the oocyte depletion in response to different chemotherapy drugs. However, cisplatin, like cyclophosphamide, induces follicular activation in mice [65,66,67]. Compelling evidence from Bolcun-Filas and colleagues indicates that primordial oocytes expressing TAp63 with a mutation at its CHK2 phosphorylation site (S621A) are not resistant to cisplatin and mafosfamide (an analogue of cyclophosphamide). In addition, studies in transgenic mice confirm that p53 contributes to primordial oocyte elimination induced by cisplatin and mafosfamide. Deleting p53 in a TAp63S621A-mutant background results in ovarian reserve resistance to cisplatin treatment [68]. In line with this result, in mice we detected CHK2-mediated phosphorylation of p53 into the nucleus of damaged oocytes following cyclophosphamide ([50] supplementary information) or following treatment with other chemotherapeutic compounds (manuscript in preparation). In addition, ChIP experiments performed on ovaries collected from treated mice demonstrate that chemotherapy-induced death of oocytes require p53 transcriptional activity ([50] supplementary information; manuscript in preparation). Together, these data from different labs reveal a critical role for p53 in oocyte response to different chemotherapeutic compounds, thus revising the model based only on TAp63alfa as the key transcription factor for DNA damage-induced ovarian reserve loss. Modifications of TAp63alfa, mediated by several kinases (like CHK2 and later by CK1), might be important for the DNA damage signaling path (possibly linked to gamma H2AX phosphorylation level in the chromatin) but not directly necessary to trigger its transcriptional activity on PUMA promoter.

## 5. Discussion

Ovarian follicle development and oocyte competence are coordinated by a very complex interplay between signaling pathways of different types of cells (oocytes, stromal cells, granulosa cells) forming the ovary. Granulosa cells play a crucial role in follicle growth, they produce hormones, and, through points of contact between cell membranes, they exchange signaling molecules and nutrition with the oocyte. Nutrition, oxygen, and hormone supply are also ensured by the vascular system, which implies the involvement of signaling pathways regulating the angiogenesis process in follicular activation. In mice, the transcription factor FOXO3 is essential in follicular development, but other key signaling molecules underlying follicle activation still remain elusive. Recent reports described multiple signaling pathways underlying primordial follicle activation and maintenance of follicle quiescence [69,70]. Chemotherapy drugs induce primordial follicle depletion [10], but it is still debated whether this is due to accelerated follicle activation or to a direct activation of DDR in the nucleus of reserve oocytes. In vivo, following cyclophosphamide treatments of mice, we show that reserve oocytes are positive for both p-Akt/p-FOXO3 and p-ATM/γH2AX expression ([50] see supplementary information). This indicates that an early DDR event is concomitant with the presence of an initial follicular activation marker (i.e., p-FOXO3). This finding may suggest that oocytes have lost their follicular quiescence to initiate the DDR. It is likely that the signaling pathways underlying ovarian follicle activation act in parallel with the activation of DDR. Thus, the primordial follicle (pre-granulosa cells plus the oocyte) may act as a single unique signaling component. A better understanding of the crosstalk between follicle reserve activation pathways and DDR will have an impact on the treatment of infertility, providing new molecular targets for clinical intervention.

## Data Availability

Not Applicable.
